# Comparative Evaluation of Electronic Acupuncture Pen and 2% Lignocaine Gel as an Intraoral Topical Anesthetic Agent in Children

**DOI:** 10.7759/cureus.47652

**Published:** 2023-10-25

**Authors:** Pranjali V Deulkar, Nilesh V Rathi, Nilima Thosar, Sphurti P Bane, Meghana A Deshpande

**Affiliations:** 1 Pediatric Dentistry, Dr. D. Y. Patil Dental College and Hospital, Dr. D. Y. Patil Vidyapeeth, Pune, IND; 2 Pediatric and Preventive Dentistry, Sharad Pawar Dental College and Hospital, Datta Meghe Institute of Higher Education and Research, Wardha, IND; 3 Pediatric Dentistry, Private Practice, Mumbai, IND; 4 Pediatric and Preventive Dentistry, Nair Dental college and Hospital, Mumbai, IND; 5 Pediatric Dentistry, Private Practice, Delhi, IND

**Keywords:** transcutaneous electrical nerve stimulation, topical anesthetic gel, pain, acupuncture pen, lignocaine

## Abstract

Background

The most exasperating aspect for pediatric patients in a dental setup is the fear and anxiety caused by injections, called "blenophobia". There are numerous local anesthetic agents available to reduce the needle prick pain. Taking into consideration the paradigm shift, there is always a possibility for alternate treatment options. This study aimed to evaluate and compare the effectiveness of transcutaneous electrical nerve stimulation (TENS) application through an electronic acupuncture pen (Meridian Energy Acupuncture Pen W-912 GENERIC) and 2% lignocaine gel as an intraoral topical anesthetic agent in children.

Method

Patients aged 6 to 12 years were eligible for inclusion. The topical anesthetic agents were administered to the patients in a bilateral split-mouth technique. In Group A, 2% topical anesthetic gel was administered on the first visit on one side of the mandible and topical anesthesia through the electronic acupuncture pen was administered on the next visit, on the opposite side of the mandible. The electronic acupuncture pen was applied on one side of the mandible on the first visit, and on the next appointment, 2% topical anesthetic gel on the other side was administered in Group B. Sound, eye, motor scale (SEM) and faces pain scale-Revised (FPS) were used as tools of evaluation after local anesthesia was administered.

Results

The comparison between electronic acupuncture pen and 2% lignocaine gel using the SEM scale shows a statistically insignificant difference (p-value = 0.082). Similarly, a comparison of FPS values between both groups indicates no significant difference (p-value = 0.582). However, results show a reduced pain perception in both groups.

Conclusion

Topical anesthetic agents are commonly used to reduce needle prick pain in children. TENS through the electronic acupuncture pen, a revisited aid in scientific research, has proved its efficacy as a topical pain reduction measure during dental treatment. This device overcomes the shortcomings of the anesthetic gels and also nullifies the chances of overdosage, hypersensitivity, and disagreeable taste. Thus, this tool can be used in dental practice for the management of pain in children.

## Introduction

Pain has been an unvarying tormentor of mankind since time immemorial. In the field of pediatric dentistry, the most stressful facet of treatment for a majority of children is the fear and anxiety sourced by the dental surroundings, predominantly to the injection referred to as "needle-phobia" or "blenophobia" [1]. Alleviating this pain in pediatric patients may aid in presenting general comfort and well-being throughout dental treatment. Pediatric dentists are persistently in search of techniques, which may present a more relaxing dental experience [2].

Topical anesthetic agents are widely used in dentistry before administering any intraoral injection. It has been proven that topical anesthetic agents have an inherent role before injections to decrease the pricking pain sensation of the needle stick [2]. To date, there are many effective and potent topical anesthetics agents, on which a number of studies have been conducted with comparative and conflicting results [2,3]. However, continuous technological research paves the way for newer alternative methods that evoke the interest of clinicians for better results.

The use of acupuncture dates back over three thousand years. It is a traditional Chinese medical technique and the world's third most common method for treating pain. There are various types of acupuncture techniques, out of which electro-acupuncture is one of the promising methods [3]. The technique involves electrical stimulation of a point with acupuncture needles on the body and then connecting the electro-machine to stimulate the point to have its effect.

However, pain due to needle insertion persists. So, the application of transcutaneous electrical nerve sti­mulation (TENS) to control pain is being explored in dentistry. In TENS therapy, pulsed electrical current is generated by alternating or by the direct current which is carried across the intact skin/mucosal surfaces via electrodes to stimulate superficial nerves for locali­zed pain relief [4].

With the advent of these techniques, the electronic acupuncture pen has been devised. The electronic acupuncture pen is a non-invasive method for producing local analgesia, and thus, this study was planned to evaluate the effectiveness of electronic acupuncture pen and 2% lignocaine gel as an intraoral topical anesthetic agent in children.

## Materials and methods

The study design and methodology were approved by the Institutional Ethical Committee, DMIMS(DU)/IEC/Aug 2019/8294. The sample size(N) was calculated using Epi Info for a bilateral split-mouth, cross-over study which was 86. However, 100 participants were taken to avoid dropouts.

Inclusion and exclusion criteria

The participants included in the study were those living in the Wardha district, who 1) were aged 6-12 years; 2) required bilateral mandibular local anesthesia as local infiltration; 3) were not exposed to any dental treatment procedure earlier; and 4) were rated grade 3 or 4 in Frankl's behavior scale. The patients who 1) exhibited any acute infections or systemic diseases; 2) were specially abled; 3) were allergic to local anesthesia; 4) had any neuromuscular disorders; and 5) had undergone any prior dental treatment were excluded.

Study design

The procedure and associated discomforts or risks were fully explained to the accompanying guardian, and their written informed consent was obtained before beginning the procedure. The participants were divided randomly into Group A and Group B by the lottery method. The area for application of the local anesthetic agent was the buccal vestibule adjacent either to the mandibular first and second primary molar or premolars depending on the eruption of the teeth. Group A: The participants in Group A were administered 2% topical gel on the first visit whereas, on the second visit, topical anesthesia was administered using the electronic acupuncture pen. Group B: The participants in Group B were administered topical anesthesia using the electronic acupuncture pen and in the second appointment 2% Lignocaine gel was administered.

**Figure 1:**
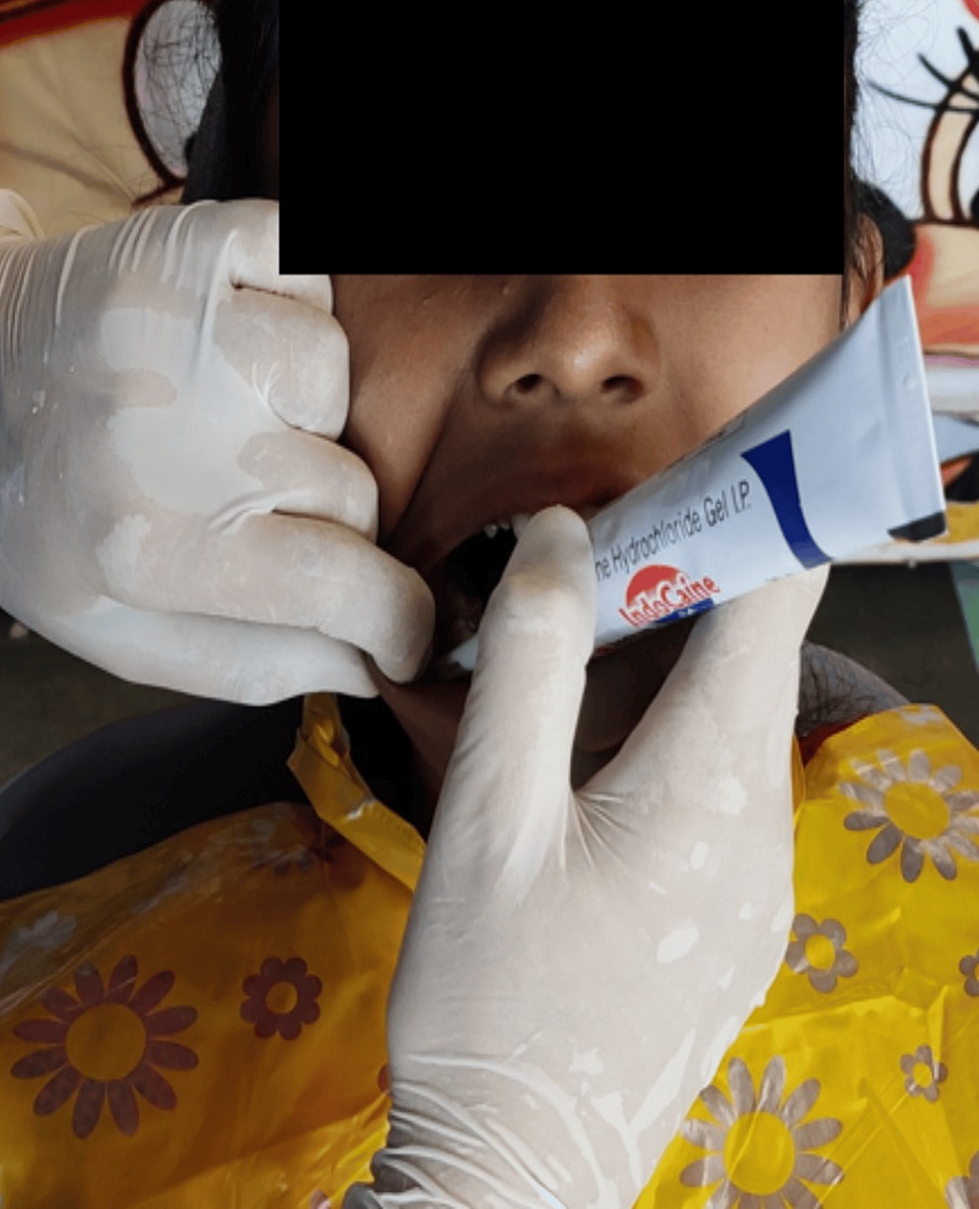
Application of the topical anesthetic gel In this figure, lignocaine topical anesthetic gel is applied in the buccal vestibule on the right side of the patient.

**Figure 2:**
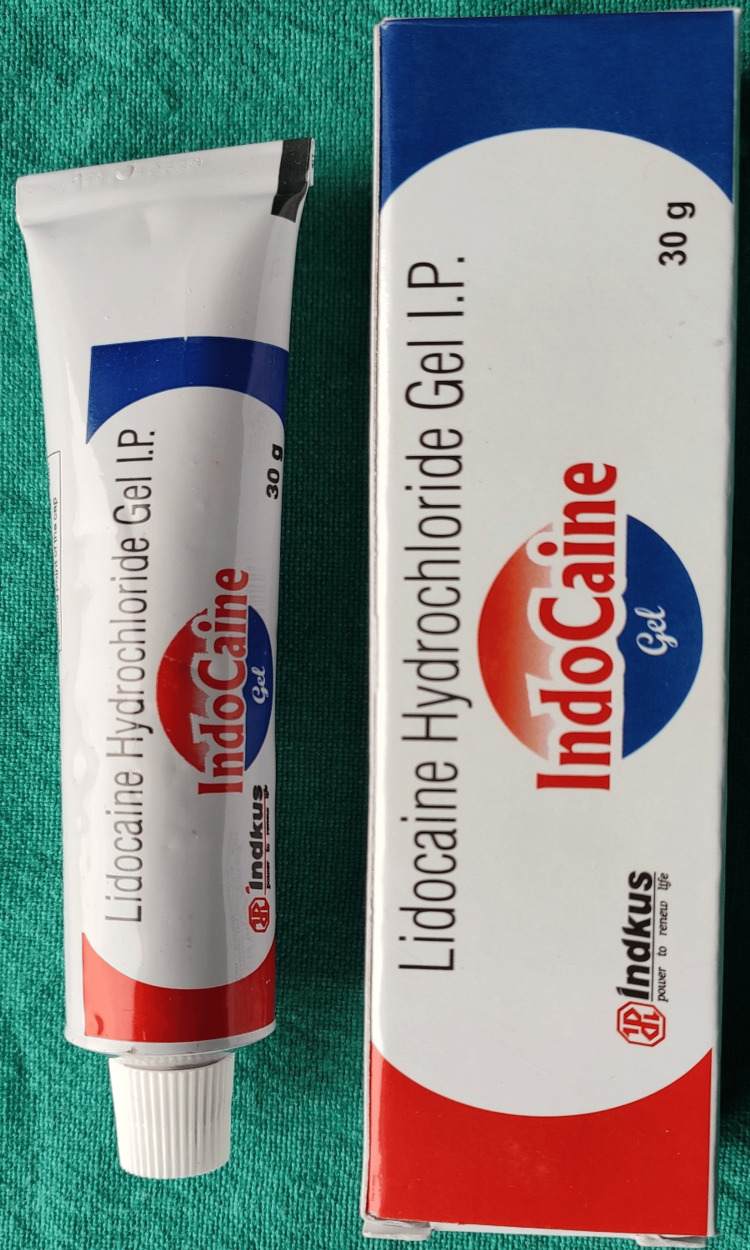
Lignocaine hydrochloride gel

The topical anesthetic agent (2% Lignocaine Hydrochloride Indocaine 2% gel INDKUS) was applied on the buccal vestibule adjacent either to the mandibular first and second primary molar or premolars using a cotton applicator tip. The tip was kept in contact with the mucosa for around two minutes (Figures 1, 2). 

**Figure 3:**
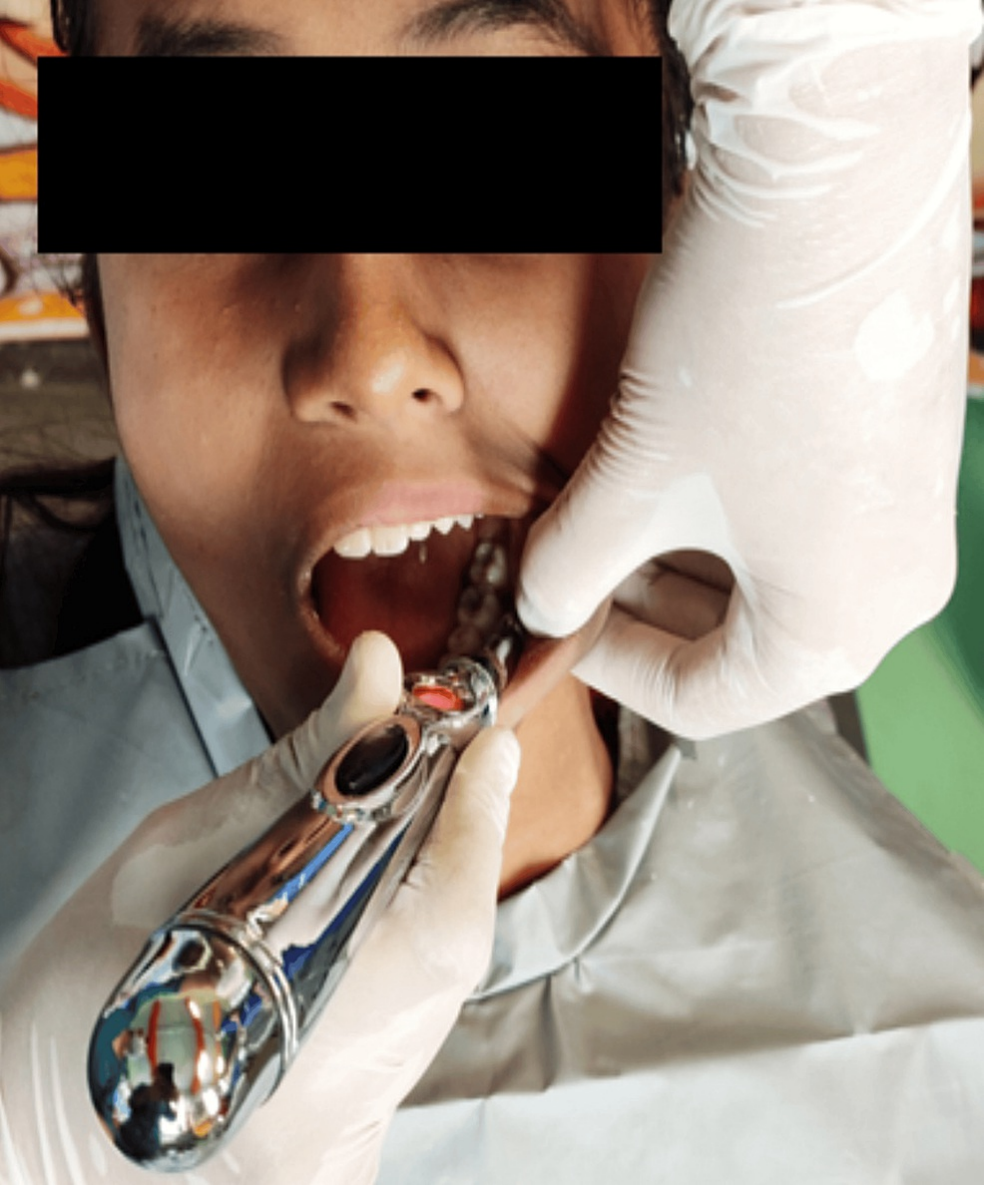
Application of TENS through the acupuncture pen In this figure, topical anesthesia is applied in the buccal vestibule on the left side using the electronic acupuncture pen. TENS: Transcutaneous electrical nerve stimulation

**Figure 4:**
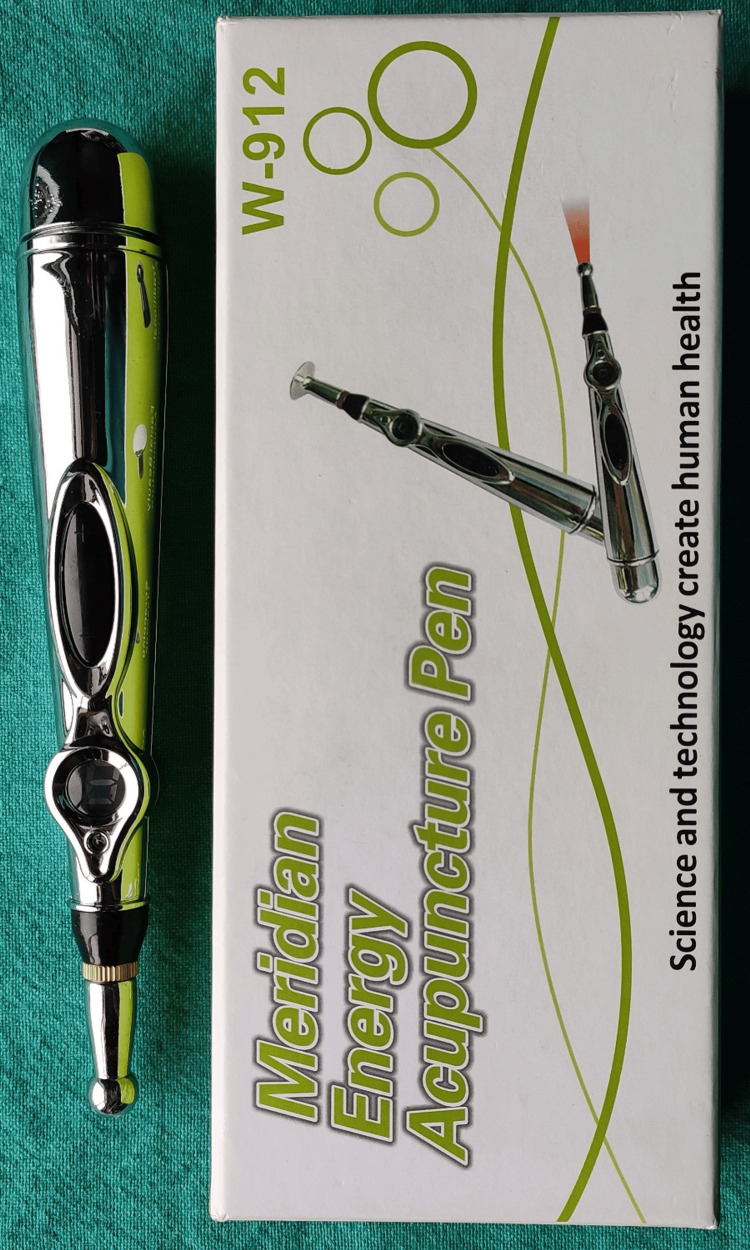
TENS meridian acupuncture pen TENS: Transcutaneous electrical nerve stimulation

The electronic acupuncture pen (Meridian Energy Acupuncture Pen W-912 GENERIC) was adjusted at a setting of '3' as instructed by the manufacturer. It was then placed over the buccal mucosa of the region where local anesthesia was to be administered. The device was in contact with the buccal mucosa only. The control over the wireless pen was switched on which was indicated by the red light on it. While keeping it in contact with the buccal mucosa, small circular motions were made for two minutes in the same region (Figures 3, 4).

After both the topical anesthetic agents were applied according to groups, local anesthesia was administered to the patients using a 26-gauge needle (Dispovan), which was inserted until bone contact was achieved and then the anesthetic agent was deposited slowly at a pace of 1 ml/minute. During needle penetration, the assessment was carried out using the Sound, Eye, Motor Scale (SEM) by two trained observers [5]. The values of this scale range from 1 to 4, with 1 as comfortable, 2 as mild discomfort, 3 as moderate discomfort, and 4 as severe discomfort. After the procedure was over, the child was shown the Wong-Baker Facial Pain Scale (FPS) and was asked to rate his/her experience. The FPS is pictographic and consists of evenly placed five faces starting from zero and going up to ten in increasing order of their discomfort [5].

Statistical analysis

The data was recorded and IBM SPSS Statistics for Windows, Version 24 (Released 2016; IBM Corp., Armonk, New York, United States) was used for the analysis. p-value <0.05 was considered as the level of significance. The normality of continuous data was analyzed by using descriptive ANOVA. The independent sample t-test and paired sample t-test were used to check the mean differences.

## Results

Table 1 shows the comparison between the mean S, E, and M values of the electronic acupuncture pen (Group 1) and 2% lignocaine gel (Group 2). The mean SEM values for Group 1 and Group 2 are 1.93±0.70 and 1.73±0.70 respectively. These values indicate a decrease in the pain perception of both groups. However, the difference is not statistically significant (p-value = 0.082). In Table 2 the comparison between the mean FPS values of the electronic acupuncture pen (Group 1) and 2% lignocaine gel (Group 2) is depicted. The mean FPS values for Group 1 and Group 2 are 3.40±1.18 and 3.40±1.180, respectively. The values indicate no significant difference in mean values between the groups (p-value = 0.582). Though the values are not statistically significant, the efficacy of the electronic acupuncture pen was more than that of 2% lignocaine gel according to both scales.

**Table 1:** Comparison of SEM (Sound, Eye, and Motor) scale between two groups This table shows the comparison between the mean S, E, and M values of the electronic acupuncture pen and 2% lignocaine gel. The values indicate no significant difference in mean (p-value = 0.082) values between the groups. NS: Not significant

Group	Mean + Standard Deviation	p-value
Electronic acupuncture pen group	1.93±0.70	0. 082 (NS)
Lignocaine gel group	1.73±0.70	

**Table 2:** Comparison of FPS (Wong-Baker facial pain scale) between two groups This table shows comparison between the mean FPS values of the electronic acupuncture pen and 2% lignocaine gel. The values indicate no significant difference in mean (p-value = 0.582) values between the groups. NS: Not significant

Group	Mean + Standard Deviation	p value
Electronic acupuncture pen group	3.40±1.18	0.582 (NS)
Lignocaine gel group	3.26±1.16	

## Discussion

Pain is an unpleasant sensory and emotional experience associated with actual or potential tissue damage or described in terms of such damage [6]. A child is exposed to the needle prick pain, which further boosts the child's anxiety and fear leading to refusal of treatment. The sight along with the application of local anesthetic injection frequently aggravates discomfort and has been illustrated as a major anxiety-provoking procedure in dentistry [7]. Topical anesthesia is an essential requirement before any pediatric dental procedures as its application before anesthesia reduces pricking pain due to needle insertion. Topical anesthetics are available in gel, spray, and ointment form, inducing a transitory loss of sensation up to a depth of 2-3 mm [8].

A systematic review and meta-analysis stated that 2% of lignocaine compounds were more effective than 20% benzocaine in decreasing pain [9]. A comparison of 20% benzocaine gel, 2% lignocaine gel, and placebo as topical anesthetics reported that both gels were equally effective in reducing the pain caused by the insertion of needles into the tissues better than the placebo paste [10]. Owing to these studies, 2% lignocaine gel was used in our study as it is considered a gold standard agent (Figures 1-3).

The major drawback of commercially available topical anesthetic gels is their inadequacy to provide bioadhesiveness to the oral mucosa due to which the targeted area may or may not receive the required amount of agent, making its effect insufficient. It has a disagreeable taste causing discomfort to the patient [11]. Other adverse responses such as overdose, hypersensitivity, idiopathic swelling of soft tissue, and anaphylaxis to the topically applied local anesthetics may be anticipated [12].

Historically, Voll developed an electrical stimulation (ES) device for application on the acupoints and meridians, thus instituting a technique known as "electroacupuncture." TENS is one such electrical stimulation [13] which is further classified into three types i.e. 1. Conventional TENS 2. Acupuncture-like TENS (AL-TENS) and 3. Intense TENS [1]. Based on the principle of AL-TENS, the electronic acupuncture pen has been devised (Figure 2). It is a non-invasive method to administer topical anesthesia through which the electrical impulses pass and pain relief is achieved. Nevertheless, it can also be applied directly to the site of needle penetration [14].

In the present study, we found that 2% lignocaine gel was less effective as compared to the electronic acupuncture pen as depicted in Tables 1, 2. However, the results were statistically insignificant. This may be owed to a varied mechanism of action of the electronic acupuncture pen.

The anesthetic effect of the electronic acupuncture pen is attributed to two key theories: The gate control theory of pain and the endogenous opioid theory. The gate control theory of pain initiated by Melzack and Wall put forth that substantia gelatinosa is a modulatory center for afferent patterns from peripheral fibers, controlling the gate system. Pain transmission occurs by small unmyelinated C fibers and their action maintains the gate open [15]. When the electronic acupuncture pen is applied (Figure 1), pain control is attained by raising the large fiber input and reducing the small fiber input leading to the closure of the gate. In 1969, Reynolds explained that endorphins are released due to electrical excitation of a periaqueductal grey region of the midbrain. Hence, the excitation of local circuits within the spinal cord is another explanation for the reduction of pain [16].

This diverse mechanism of action possibly explains the better results seen in the electronic acupuncture pen group. This is in support of research carried out by Meechan et al. recommending that using electrical stimulation lessens injection distress better during nerve block administration when compared to 2% lignocaine [17].

Pain assessment is a very individualized and subjective occurrence. After the administration of local anesthesia, the Wong-Baker FPS was described to the children for a subjective pain assessment [16]. However, objective assessment is also important, as the child's perception may sometimes be misleading. The SEM scale was used to evaluate the objective pain experienced by each child.

The use of an electronic acupuncture pen reduces anxiety when used on the acupoint. This non-invasive application does not cause any adverse reactions and is comparatively cost-effective. The electronic acupuncture pen can be employed to replace 2% lignocaine gel, as its use may give better results in pain alleviation during needle pricking. The application of this electronic acupuncture pen was only done in adherent mucosa for local infiltration. However, the study has limitations in assessing objective parameters like heart rate, oxygen saturation, blood pressure, etc. Such parameters would have been helpful in deriving the efficacy of the TENS in a better manner.

## Conclusions

Topical anesthetic agents are commonly used to reduce needle prick pain in children. TENS through electronic acupuncture pen, a revisited aid in scientific research has proved its efficacy as a topical pain reduction measure during dental treatment. This device overcomes the shortcomings of the anesthetic gels and also nullifies the chances of overdosage, hypersensitivity, and disagreeable taste. Thus, this tool can be used in dental practice for the management of pain in children.
